# A vagal route to memory: evidence from invasive and non-invasive electrical vagus nerve stimulation studies and areas for future clinical application

**DOI:** 10.3389/fnhum.2025.1595737

**Published:** 2025-07-08

**Authors:** Christoph Szeska, Carlos Ventura-Bort, Manon Giraudier, Mathias Weymar

**Affiliations:** ^1^Department of Biological Psychology and Affective Science, Faculty of Human Sciences, University of Potsdam, Potsdam, Germany; ^2^Faculty of Health Sciences Brandenburg, University of Potsdam, Potsdam, Germany

**Keywords:** vagus nerve stimulation, emotional memory, associative memory, fear extinction, Alzheimer’s disease, PTSD - Posttraumatic stress disorder, anxiety disorders, mild cognitive impairment - MCI

## Abstract

The ability to remember emotionally significant stimuli and stimulus associations is critical to survival, as it ensures that rewarding and threatening events can be recalled to guide future behavior. Consequently, events are consolidated more strongly into long-term memory as they are encoded under heightened emotional arousal. Such memory prioritization is partly driven by the release of peripheral adrenaline, which acts as a bodily signal emphasizing an event’s emotional significance and enhances plasticity in the brain. Animal research suggest that the vagus nerve translates elevated peripheral adrenaline into central noradrenergic activation of memory-relevant brain areas via its projections to the brainstem locus coeruleus–the main source of noradrenaline in the brain. The possibility of vagus nerve stimulation (VNS), both invasively (iVNS) and non-invasively (i.e., transcutaneously; tVNS), has opened up new avenues to test a potential vagal route to memory in humans whilst circumventing the necessity of actual peripheral adrenergic release. Here, we briefly review recent research applying iVNS and tVNS in a variety of animal and human emotional episodic memory and Pavlovian conditioning and extinction learning experiments, supporting a critical role of the vagus nerve in modulating emotional memories. Based on this body of evidence, we highlight clinical areas where VNS may therefore serve as an adjunct to treatments for neurocognitive, anxiety- and trauma-related disorders, that aim at improving learning and memory consolidation. In fact, a brief review of (sub-) clinical studies shows that VNS alleviates symptoms in mild cognitive impairment, Alzheimer’s disease as well as anxiety- and trauma-related disorders.

## Introduction

Memories are created through a highly selective filter: While mundane experiences easily fade away, events laden with emotional salience are etched deeply into our remembrance (Dolan, 2002; LaBar and Cabeza, 2006; Wang and Bukuan, 2015; Rouhani et al., 2023). Emotions are typically elicited during events that are critical to our survival and can therefore functionally be conceived as tags emphasizing the motivational significance of stimuli and stimulus associations (Bradley et al., 2001b). Thus, memory prioritization for emotional material is highly adaptive, as it ensures that significant events (e.g., receiving rewards or facing threats) can be recalled to guide our future behavior (e.g., approach or escape) (McIntyre et al., 2012; Dunsmoor and Kroes, 2019; Szeska et al., 2022). Given its relevance, it may therefore be somewhat surprising that the memory-enhancing effect of emotion has been recognized for a long time (James, 1890), yet the underlying mechanisms of action have only begun to be uncovered in the second half of the last century (Easterbrook, 1959).

More than 50 years later, it is now well established that memory consolidation for emotional experiences is prioritized as they are associated with a profound increase in arousal, entailing increased attentional and perceptual processing (Bradley et al., 2001a; LaBar and Cabeza, 2006; Rouhani et al., 2023). For instance, pictures that have been rated as highly arousing are overall better remembered than low arousing ones, as indicated by increased free recall (Bradley et al., 1992; Hamann et al., 1997) and high-confidence (recollection-based) recognition memory (Dolcos et al., 2005, 2020; Weymar et al., 2009). Even non-emotional pictures of tools or animals have found to be preferably consolidated if they have been associated with physiologically arousing stimuli, e.g., an aversive electric shock or a monetary reward (Dunsmoor et al., 2015a; Patil et al., 2017). Accordingly, dedicated strategies that elevate arousal around the time of stimulus encoding, e.g., by stressful tasks, have found to additionally foster memory enhancement for emotional material (Nielson et al., 1996; Nater et al., 2007; Schwabe et al., 2008; Weymar et al., 2012), while strategies that decrease arousal, e.g., by relaxing music, have shown to attenuate such effect (Rickard et al., 2012; for a review see McGaugh, 2018).

**FIGURE 1:**
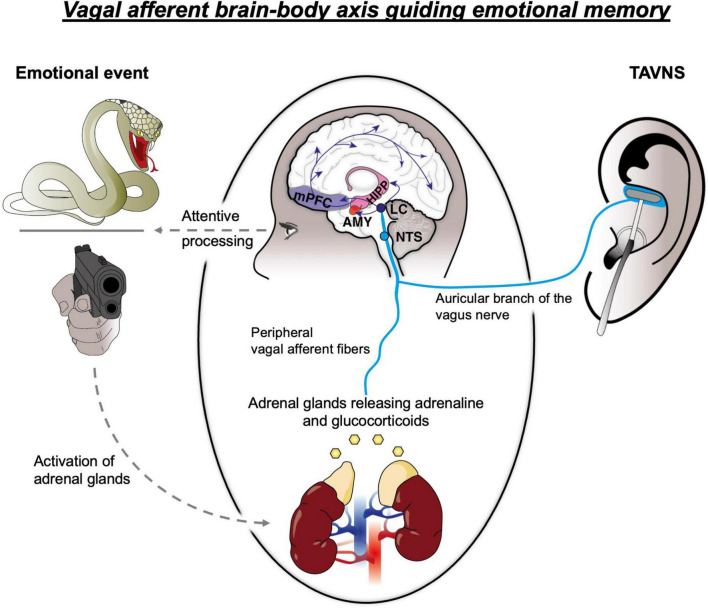
Schematic representation of interacting neural systems presumed to drive arousal-based memory enhancement, involving the adrenal glands, vagal afferent fibers and multiple brain regions, including the nucleus of the solitary tract (NTS), locus coeruleus (LC), amygdala (AMY), hippocampus (HIPP) and medial prefrontal cortex (mPFC). TAVNS, transcutaneous auricular vagus nerve stimulation.

Extensive research in animals and humans unveiled that arousal-based memory enhancement is hinged upon the adrenal glands’ immediate adrenaline and delayed glucocorticoids release (Roozendaal et al., 2009; McIntyre et al., 2012; McGaugh, 2018). For instance, aversive emotionally arousing pictures (e.g., of a snake or a gun pointing towards the participant; see Figure 1) lead to a profound increase in peripheral adrenergic activation, as indexed by endocrine markers such as salivary alpha amylase (sAA), and cortisol release, both of which positively covarying with enhanced memory retention (Abercrombie et al., 2003; Codispoti et al., 2003; Van Stegeren et al., 2006; Segal and Cahill, 2009). Highlighting the particular role of adrenergic activation in memory consolidation, depleted levels of peripheral adrenaline–e.g., due to adrenalectomy–in contrast impair memory performance for emotional stimuli (Borrell et al., 1983). Providing even stronger mechanistic evidence, small doses of exogeneous adrenaline foster memory enhancement for emotional material and even reverse the effects of adrenalectomy (Borrell et al., 1983; Cahill and Alkire, 2003), while beta-blockers that attenuate adrenergic transmission prevent memory enhancement from unfolding (Van Stegeren et al., 1998; for electrocortical evidence see e.g., Weymar et al., 2010).

Pharmacological and imaging studies have demonstrated, that increased levels of peripheral adrenaline invoke such memory enhancement by impacting on the neural transmission in the amygdala–a central hub organizing the establishment of emotional memories via its projections to the hippocampus and cortical regions (Dolcos et al., 2004; Kensinger, 2004; Ritchey et al., 2008; McIntyre et al., 2012; McGaugh, 2018). Accordingly, the activity of this region during encoding of emotionally arousing material increases and positively correlates with memory performance (Canli et al., 2000; Figure 1), while bilateral amygdala lesions have found to prevent memory enhancement for emotionally salient stimuli (Adolphs et al., 1997; Phelps et al., 1998). At this, increases in amygdala activity are indeed strikingly concomitant to increases in adrenergic activity (Van Stegeren et al., 2006; van Stegeren et al., 2007), suggesting that the release of adrenaline might stimulate this region to ultimately invoke memory enhancement: In line with this view, direct infusions of adrenaline into the amygdala foster, while direct infusions of beta-blockers attenuated emotional memory enhancement in animals (Liang et al., 1995).

However, as peripheral adrenaline is unable to cross the blood-brain barrier (Weil-Malherbe et al., 1959), a neural axis–the vagus nerve-has been presumed to convey information about elevated adrenergic levels from the body to the brain, thereby indirectly increasing neural transmission in memory-relevant brain regions. The vagus nerve, as a cranial nerve consisting of 80% afferent fibers, had long been considered a major autonomic communication route by which the brain receives information about the state of the inner body (Foley and DuBois, 1937; Berthoud and Neuhuber, 2000; McIntyre et al., 2012). Importantly, vagal afferents indeed innervate the adrenal glands and are highly responsive to the release of peripheral adrenaline due to a high number of beta-adrenergic receptors (Coupland et al., 1989; Niijima, 1992; Miyashita and Williams, 2006; Figure 1). Thus, the release of peripheral adrenaline is able to activate vagal afferents, which then project to the nucleus of the solitary tract (NTS) in the brainstem where adrenergic activation is finally synapsed onto the main hub of noradrenaline in the brain: the locus coeruleus (LC) (Miyashita and Williams, 2004; McIntyre et al., 2012; McGaugh, 2018; Figure 1). By way of the vagus nerve, a peripheral release of adrenaline is thus translated into increased noradrenergic release via the LC-brainstem arousal system. This system ultimately projects to memory-relevant brain areas including the basolateral amygdala (AMY), hippocampus (HC) and cortex (e.g., the medial prefrontal cortex; mPFC), increasing noradrenergic transmission and, thus, promoting plasticity in these areas to eventually foster memory establishment (McIntyre et al., 2012; Mather et al., 2016; McGaugh, 2018; Figure 1). Accordingly, the vagus nerve was presumed to constitute the first relay of a neural circuit mediating the memory enhancing effects of emotional arousal. Indeed, animal research supports this concept by showing that an invasive stimulation of the vagus nerve (iVNS) elicits firing in the NTS and LC and eventually increases noradrenergic transmission in the amygdala (Hassert et al., 2004; Hulsey et al., 2017; Cooper et al., 2021). Consequently, iVNS in fact promotes the memory formation in animals (Clark et al., 1998) while such memory enhancement is prohibited from unfolding, when the LC is optogenetically silenced (Calderon-Williams et al., 2024).

While a major role of the vagus nerve in the formation of emotional memories is thus supported by the animal model (see for a review Olsen et al., 2023), a comparable role has long been elusive in humans. In 1999, however, Clark and colleagues demonstrated that iVNS in fact leads to an enhancement in word recognition memory (Clark et al., 1999). Despite such promising preliminary evidence, though, subsequent stimulation studies that further explored a vagal route to memory in humans remained scarce due to the requirement of invasive surgery. This changed at the beginning of the millennium, when Peuker and Filler (2002) discovered that vagal afferents reach the body’s surface at the *Cymba Conchae* of the human auricle – a skin area that is exclusively innervated by the auricular vagal branch (Figure 1), allowing non-invasive vagal stimulation (transcutaneous auricular vagus nerve stimulation; taVNS) with minimal side effects (Giraudier et al., 2025). TaVNS leads to activation in the vagal afferent network important for memory formation including the NTS, LC, amygdala, hippocampus and mPFC, and also results in an increase in central noradrenergic transmission (Frangos et al., 2015; Yakunina et al., 2017; Ventura-Bort et al., 2018; Sclocco et al., 2019; Borgmann et al., 2021; Teckentrup et al., 2021; Giraudier et al., 2022; Figure 1). The availability of this neuromodulatory strategy has opened up new possibilities to test whether the vagus nerve fulfils a similar role for emotional memory in humans as it does in animals. In the following sections, we will provide a brief overview of recent stimulation studies, which support the view that vagal firing promotes episodic and associative emotional memory establishment. Based on this body of evidence, we will highlight potential areas of clinical application where VNS may be utilized to facilitate the long-term consolidation of learning experiences.

## The role of the vagus nerve in episodic memory

Initial evidence for the causal influence of ascending vagal fibers in emotional memory came from animal research (Clark et al., 1995, 1998). In one study, Clark et al. (1998) trained rats in an inhibitory avoidance task, where animals were to learn to avoid an electric shock. Directly after, animals received either 30 s of VNS or sham stimulation (see Table 1). The authors observed that VNS compared to sham stimulation improved memory performance, assessed 24 h after stimulation, especially for intermediate stimulation intensities (Clark et al., 1998; see also Sanders et al., 2019; Olsen et al., 2022; for a review see Olsen et al., 2023; also see Table 1). Subsequent studies have shown that VNS modulates molecular mechanisms within the HC, suggesting that VNS-induced memory improvements are likely related to hippocampal activity (c.f., Olsen et al., 2023).

**TABLE 1:** Overview of reviewed studies with particular emphasis on critical stimulation parameters and central effects.

					Stimulation parameters							
Authors	Topic	Subject	VNS type	VNS site	Fre-quency	Pulse width	Duty cycle	Duration per session*	Intensity	Timing	Task	VNS effect
Clark et al., 1995	Episodic memory	Animal	iVNS	Left	20 Hz	500 μs	continuous	30 s	0.2/0.4/0.8 mA	After encoding	Inhibitory avoidance	Improved memory retention for 0.4 mA of stimulation
Clark et al., 1998	Episodic memory	Animal	iVNS	Left	20 Hz	500 μs	continuous	30 s	0.2/0.4/0.8 mA	After encoding	Inhibitory avoidance	Improved memory retention for 0.4 mA of stimulation
Sanders et al., 2019	Episodic memory	Animal	iVNS	Left	30 Hz	100 μs	Intermittent 0.5 s trains	Effective stimulation duration ∼ 51 s	0.8 mA	During multiple encoding sessions	Object recognition	Increased novelty preference as an index of improved memory retention
Clark et al., 1999	Episodic memory	Human (epileptic patients)	iVNS	Left	30 Hz	500 μs	continuous	30 s	0.50 mA, 0.75 –1.50 mA	After encoding	Word recognition	Improved recognition memory for 0.50 mA of stimulation
Olsen et al., 2022	Episodic memory	Animal	iVNS	Left	30 Hz	100 μs	Intermittent 0.5 s trains	Effective stimulation duration ∼ 50 s	0.8 mA	During encoding	Inhibitory avoidance and object recognition	Improved memory performance in both tasks
Mertens et al., 2022	Episodic memory	Human (epileptic patients)	iVNS and taVNS	Left	30 Hz	500 μs	continuous	30 s	invasive 0.5/1.0 mA (increased by 0.125 and 0.25 mA throughout treatment) non-invasive: individually adjusted	After encoding	Word recognition	No immediate memory improvement. However, improved memory retention after 6 weeks of VNS
Ghacibeh et al., 2006	Episodic memory	Human (epileptic patients)	iVNS	Left	No report	No report	continuous	30 s	0.5 mA	During encoding	Word recall	Improved memory retention
Ventura-Bort et al., 2021	Episodic memory	Human	taVNS	Left	25 Hz	250 μs	continuous	7 min	individually adjusted	During encoding	Picture recognition	Improved high-confidence recognition memory for emotional pictures | Enhanced electrocortical correlates of emotional encoding and memory retrieval
Ludwig et al., 2025	Episodic memory	Human	taVNS	Left	25 Hz	250 μs	Intermittent 3 s trains	Effective stimulation duration 2.4 min	3.0 mA/5.0 mA	During encoding	Picture recognition	Improved recognition memory for emotional pictures
Ventura-Bort et al., 2025 (Exp. 1)	Episodic memory	Human	taVNS	Left	25 Hz	250 μs	Intermittent 30 s trains	Effective stimulation duration 7.5 min	individually adjusted	During and after encoding	Picture recognition	No effect on memory performance | Enhanced electrococortical correlates of emotional encoding and memory retrieval in taVNS condition
Ventura-Bort et al., 2025 (Exp. 2)	Episodic memory	Human	taVNS	Left	25 Hz	250 μs	Intermittent 30 s trains	Effective stimulation duration 7.5 min	individually adjusted	During encoding	Picture recognition	Improved high-confidence recognition memory for emotional pictures | Enhanced electrocortical correlates of emotional encoding and memory retrieval
Giraudier et al., 2020	Episodic memory	Human	taVNS	Left	25 Hz	250 μs	Intermittent 30 s trains	Effective stimulation duration 11.5 min	individually adjusted	Before, during and after encoding	Word recognition	Improved high-confidence recognition memory for neutral and emotional words
Mertens et al., 2020	Episodic memory	Human	taVNS	No report	25 Hz	250 μs	continuous	30 s	0.5 mA and individually adjusted	After encoding	Word recognition	No effect of taVNS
Peña et al., 2013	Associative memory	Animal	iVNS	Left	20 Hz	500 μs	intermittent 30 s trains	Effective stimulation duration 2 min	0.4 mA	During extinction	Single-cue fear conditioning and extinction	Improved fear extinction learning and memory retention
Peña et al., 2014	Associative memory	Animal	iVNS	Left	30 Hz	500 μs	intermittent 30 s trains	Effective stimulation duration 2 min	0.4 mA	During extinction	Single-cue fear conditioning and extinction	Improved between-session extinction/equivalent to extended extinction
Alvarez-Dieppa et al., 2016	Associative memory	Animal	iVNS	Left	20 Hz	500 μs	intermittent 30 s trains	Effective stimulation duration 2 min	0.4 mA	During initial extinction	Single-cue fear conditioning and extinction	Improved between-session extinction
Noble et al., 2017	Associative memory	Animal (PTSD model)	iVNS	Left	20 Hz	100 μs	intermittent 30 s trains	Effective stimulation duration 2 min	0.4 mA	During multiple extinction sessions	Single-cue fear conditioning and extinction	Improved between-session extinction, reversal of extinction impairments and attenuation of PTSD-like symptoms due to prior prolonged stress
Noble et al., 2019	Associative memory	Animal	iVNS	Left	20 Hz	100 μs	intermittent 30 s trains	Effective stimulation duration 2 min	0.4 mA	During multiple extinction sessions	Single-cue fear conditioning and extinction	Improved between-session extinction; Improved extinction memory generalization; Inherent anxiolytic effects of VNS
Souza et al., 2019	Associative memory	Animal (PTSD model)	iVNS	Left	30 Hz	100 μs	intermittent 30 s trains	Effective stimulation duration 2.5 min	0.4 mA	During multiple extinction sessions	Fear conditioning and extinction	Improved between-session extinction, reduced fear renewal and inherent anxiolytic effects of VNS
Souza et al., 2021	Associative memory	Animal (PTSD model)	iVNS	Left	30 Hz	100 μs	intermittent 0.5 s trains	Effective stimulation duration 10 s	0.4/0.8/1.6 mA	During multiple extinction sessions	Single-cue fear conditioning and extinction	Improved between-session extinction at 0.4 and 0.8 mA, improved long-term extinction retention at 0.8 mA, no extinction enhancement at 1.6 mA
Souza et al., 2022	Associative memory	Animal	iVNS	Left	30 Hz	100 μs	intermittent 0.5 s trains	Effective stimulation duration up to 40 s	0.5/0.8 mA	During multiple extinction sessions	Single-cue fear conditioning and extinction	Strong vs. modest between-session extinction improvements when stimulation was paired vs. unpaired with CS, respectively
Calderon-Williams et al., 2024	Associative memory	Animal	iVNS	Left	30 Hz	500 μs	intermittent 2 s trains	Effective stimulation duration 32 s	0.8 mA	During initial extinction	Single-cue fear conditioning and extinction	Facilitated between-session extinction
Childs et al., 2017	Associative memory	Animal	iVNS	Left	30 Hz	100 μs	intermittent 0.5 s trains	Unclear	0.8 mA	During multiple extinction sessions	Extinction of cocaine seeking	Facilitated between-session extinction and reduced reinstatement of cocaine seeking
Childs et al., 2019 (Exp. 1)	Associative memory	Animal	iVNS	Left	30 Hz	500 μs	Intermittent 30 s trains	Effective stimulation duration 3 min	0.4 mA	During or after multiple extinction sessions	Conditioned place preference and extinction	Reduced reinstatement of conditioned place preference for cocaine
Childs et al., 2019 (Exp. 2)	Associative memory	Animal	iVNS	Left	30 Hz	500 μs	Intermittent 30 s trains	Unclear	0.4 mA	During multiple extinction sessions	Extinction of cocaine seeking	Enhanced extinction from drug seeking, reduced context- and cue-induced reinstatement of cocaine seeking
Burger et al., 2016	Associative memory	Human	taVNS	Left	25 Hz	No report	Intermittent 30 s trains	Unclear	0.5 mA	During initial extinction	Differential-cue fear conditioning and extinction	Facilitated within-session extinction (threat expectancy), no effect on extinction recall
Burger et al., 2017	Associative memory	Human	taVNS	Left	25 Hz	250 μs	Intermittent 30 s trains	Effective stimulation duration 10 min	0.5 mA	During initial extinction	Differential-cue fear conditioning and extinction	Facilitated within-session extinction (threat expectancy), no effect on extinction recall
Burger et al., 2018	Associative memory	Human	taVNS	Left	25 Hz	250 μs	Intermittent 30 s trains	Effective stimulation duration ∼ 13 min	0.5 mA	During initial extinction	Differential-cue fear conditioning and extinction	No effect of taVNS
Szeska et al., 2020	Associative memory	Human	taVNS	Left	25 Hz	250 μs	Intermittent 30 s trains	Effective stimulation ∼ 4 min	individually adjusted	During initial extinction	Single-cue fear conditioning and extinction	Facilitated within-session extinction and between-session extinction (threat expectancy, startle response)
Szeska et al., 2021	Associative memory	Human	taVNS	Left	25 Hz	250 μs	Intermittent 30 s trains	Effective stimulation ∼ 4 min	individually adjusted	During initial extinction	Single-cue fear conditioning and extinction	Facilitated within-session extinction (heart rate)
D’Agostini et al., 2025	Associative memory	Human	taVNS	Left	25 Hz	250 μs	continuous	Effective stimulation duration ∼ 15 min	individually adjusted	During initial extinction	Differential-cue fear conditioning and extinction	No effect of taVNS
Genheimer et al., 2017	Associative memory	Human	taVNS	Left	25 Hz	250 μs	Intermittent 30 s trains	Effective stimulation duration ∼ 20 min	individually adjusted	Before and during initial extinction	Contextual fear conditioning and extinction	No effects of taVNS
Jacobs et al., 2015	MCI and AD	Human (elderly)	taVNS	Left	8 Hz	200 μs	continuous	Effective stimulation duration 17 min	5.0 mA	During and after encoding	Face-Name assocation task	Improved recognition memory
Murphy et al., 2023	MCI and AD	Human (MCI)	taVNS	Left	20 Hz	50 μs	continuous	Effective stimulation duration 6 min	individually adjusted	During MRI scanning	Resting State MRI	Altered functional connectivity between brain regions involved in semantic and salience processing
Wang et al., 2022	MCI and AD	Human (MCI)	taVNS	Left	20 Hz and 50 Hz	No report	continuous	Effective stimulation duration 30 min	individually adjusted	Between baseline and follow-up testing (after 24 weeks of treatment)	Battery of cognitive tests	Improved cognitive (including memory) performance
Dolphin et al., 2023	MCI and AD	Human (MCI)	taVNS	Left	Unclear	Unclear	Unclear	Unclear	Unclear	Unclear	Face-Name assocation task	Improved recognition memory
Sjogren et al., 2002	MCI and AD	Human (AD patients)	iVNS	Left	20 Hz	No report	intermittent 30 s trains	Unclear	0.25 mA (increased throughout treatment to 0.5 mA)	After baseline and during follow-up testing (at 3 and 6 months of treatment)	Battery of cognitive tests	Improved cognitive performance after 3 and 6 months of VNS treatment
Merrill et al., 2006	MCI and AD	Human (AD patients)	iVNS	Left	20 Hz	No report	intermittent 30 s trains	Unclear	0.25 mA (increased throughout treatment to 0.5 mA)	After baseline and during follow-up testing (1 year after treatment)	Battery of cognitive tests	Improved cognitive performance after 1 year of VNS treatment
Powers et al., 2025	PTSD and Anxiety disorders	Humans (treatment-resistant PTSD patients)	iVNS	Left	30 Hz	100 μs	intermittent 0.5 s trains	Unclear	0.8 mA	During prolonged exposure sessions	Repeated prolonged exposure sessions (therapist guided and alone)	Alleviation of PTSD symptoms lasting for at least 6 months, loss of PTSD diagnosis
Szeska et al., 2025	PTSD and Anxiety disorders	Humans (spider phobic individuals)	taVNS	Left	25 Hz	250 μs	Intermittent 30 s trains	Effective stimulation duration ∼ 11 min	individually adjusted	During laboratory exposure to spider pictures	Repeated *in vivo* exposures separated by one *in vitro* laboratory exposure	Inhibition of stimulus-specific threat responses (heart rate, corrugator activity), reduced avoidance behavior towards exposed tarantula

*The application duration may differ substantially from effective stimulation duration (e.g., upon long inter-stimulation intervals). Also note, that the effective stimulation duration may accumulate, if there are multiple VNS sessions per day.

In a follow up study, Clark et al. (1999) attempted to extend their animal findings to humans. In this study, epileptic patients with an implanted VN stimulator underwent verbal learning before receiving either active VNS at varying intensities (Table 1) or no stimulation in a control condition. Memory performance was tested in an immediate word-recognition test. Results showed that when the stimulation device was turned on after encoding, participants exhibited better memory performance, compared to when the stimulator remained off, particularly at intermediate stimulation intensities (Clark et al., 1999). Subsequent investigations on the effects of iVNS on memory performance have, however, yielded mixed findings. While some studies also reported positive effects of iVNS on memory performance (Ghacibeh et al., 2006), others failed to observed memory improvements after iVNS (Mertens et al., 2022; for a review see Olsen et al., 2023; see Table 1).

Unlike animal studies, which focused on emotional memories, human studies using iVNS did not assess episodic memory for emotional information, limiting the generalization of the same neural path from animals to humans. Ventura-Bort et al. (2021) therefore investigated the role of the VN on the formation and consolidation of emotional episodic memories in humans using taVNS. In this study, participants underwent two encoding sessions in which unpleasant and neutral images were encoded while receiving taVNS or sham stimulation in a counterbalanced, within-subjects design. One week later, recognition memory was tested by also assessing the contribution of familiarity vs. recollection-based remembering (i.e., *low vs. high confidence*; Wixted and Stretch, 2004), with the latter representing a more elaborate mnemonic process that particularly reflects increased amygdala and hippocampal activity (Dolcos et al., 2005, 2020).

Although no overall effects of VNS on memory performance were found (see also Ludwig et al., 2025), unpleasant images encoded under taVNS were more often retrieved with high confidence (Ventura-Bort et al., 2021), indicating a recollection-driven increase for emotional but not neutral images (see also Ludwig et al., 2025 for general memory improvements for unpleasant images encoded under taVNS). These findings were also accompanied by larger recollection-sensitive brain potentials (late ERP Old/New effect) during retrieval of emotional scenes encoded under taVNS, compared to sham stimulation (for a recent conceptual replication of the electrocortical findings using different stimulation protocols, see Ventura-Bort et al., 2025). Similar recollection-related results were also obtained in a behavioral study investigating the effects of taVNS on memory for emotional and neutral words (Giraudier et al., 2020). Although no overall effects of taVNS were found, participants receiving taVNS during the word encoding task showed a recollection-driven advantage (i.e., for words with the highest confidence) 1 day later (but see for no effects when taVNS was applied offline using a same day memory paradigm, Mertens et al., 2020). In contrast to Ventura-Bort et al. (2021), however, no emotion-specific memory enhancement was observed after taVNS (Giraudier et al., 2020), which may be partly explained by the use of the less arousing emotional material (words compared to high arousing pictures, c.f., Ventura-Bort et al. (2021)).

Altogether, these results suggest that stimulation of vagal afferents improves the formation of episodic, particularly recollection-based (i.e., hippocampal-mediated), memories, as indicated by behavioral and electrophysiological measures.

## The role of the vagus nerve in associative memory

Importantly, it is not only vital for survival to remember distinct stimuli, but also to remember associations between them, ultimately allowing to anticipate upcoming events based on past experiences. In both animals and humans, such associative emotional memory is predominantly investigated by means of Pavlovian conditioning and extinction protocols. During conditioning, an inherently neutral conditioned stimulus (CS; e.g., a light in animal research; a geometrical figure in human research) repeatedly predicts the occurrence of an emotionally salient, i.e., unpleasant or pleasant, unconditioned stimulus (US; e.g., electric shock or food incentive) (Lonsdorf et al., 2017). As a result, the CS gains the capacity to elicit conditioned emotional responses (e.g., fear in case of a highly aversive US), reflecting a learned CS**–**US association (Lonsdorf et al., 2017). In contrast, during subsequent extinction protocols, the CS is no longer paired with any US (Lonsdorf et al., 2017). Thus, a novel association (CS-No US) is established, that inhibits the activation of an originally conditioned memory trace and thus reduces conditioned emotional responses (Dunsmoor et al., 2015b). Importantly, vagal projection targets (see Figure 1) play pivotal roles in both conditioning and extinction: Plasticity in the basolateral amygdala underlies initial associative *learning* (i.e., initial conditioning and extinction), while the medial prefrontal cortex is particularly involved in the consolidation and recall of extinction memory (Herry et al., 2008; Senn et al., 2014; Tovote et al., 2015; Szeska et al., 2022). In addition, noradrenaline heavily impacts on the plasticity in these areas, and consequently increased activity of the LC-NA system has found to promote associative memory processes (Uematsu et al., 2017; Giustino and Maren, 2018).

Accordingly, animal studies found promoted associative emotional memory by iVNS: Rats, that underwent an extinction protocol under iVNS, consistently show promoted extinction of previously conditioned fear–an effect, that maintains even for 10 days and may even reverse experimentally induced extinction impairments (Peña et al., 2013, 2014; Alvarez-Dieppa et al., 2016; Noble et al., 2017, 2019; Souza et al., 2019, 2021, 2022; see Table 1). Importantly, this extinction enhancement by iVNS is abolished if the LC is optogenetically silenced (Calderon-Williams et al., 2024), providing strong mechanistic evidence that respective memory improvements are dependent to vagal projections to the LC-NA system. Notably, the enhancing effects of iVNS are not limited to the extinction of fear, but also apply to the extinction of conditioned appetitive responses (e.g., cocaine-induced place preference) (Childs et al., 2017, 2019). Animal research therefore strongly suggests, that the vagus nerve is involved in guiding the establishment of associative emotional memory, primarily through its projections to the LC-NA system.

Human research utilizing taVNS has largely translated these findings from the animal model, although results have been mixed (see e.g., Burger et al., 2018 and D’Agostini et al., 2025; Szeska et al., 2020, 2021; see Table 1): Using a differential-cue Pavlovian fear conditioning paradigm, requiring discriminative learning between a threat and a safety cue, Burger and colleagues showed that taVNS accelerates the extinction of previously conditioned fear (Burger et al., 2016, 2017). Notably, though, these extinction enhancements were limited to verbal report measures of fear (i.e., threat expectancy). Yet, in a simpler single-cue Pavlovian fear conditioning paradigm, which is more closely adapted to animal research and requires simpler associative learning (either threat learning or not in a between-group design), taVNS has found to accelerate the extinction of verbal report, behavioral and physiological components of the fear response (Szeska et al., 2020, 2021). Consistent with prolonged extinction enhancements observed in animals, such beneficial effect maintained for even 4 weeks. However, the extinction enhancements by taVNS may be limited to the learning of associations between distinct cues: In a differential-context Pavlovian fear conditioning protocol, requiring discriminative learning of a threat-signaling vs. safety-signaling environment in virtual reality, taVNS failed to promote the extinction of contextually-related conditioned responses (Genheimer et al., 2017).

As for episodic memory, the current body of evidence therefore also generally supports a pivotal role of the vagus nerve in the establishment of human associative emotional memory, although beneficial effects primarily unfold when simple cue-outcome relationships are to be learned.

## A potential role of vagus nerve stimulation in the treatment of mental disorders

**FIGURE 2:**
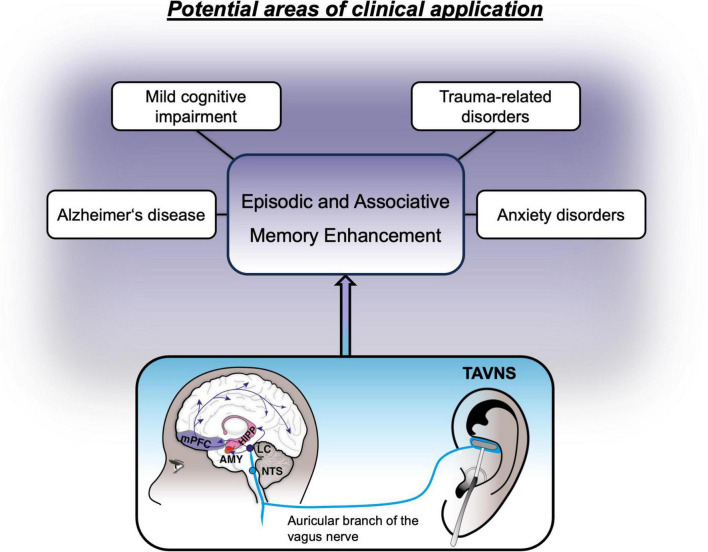
Non-exhaustive overview of potential clinical areas where treatment might benefit from vagus nerve stimulation.

Based on the beneficial effects of non-invasive VNS on memory formation, this method was rendered a potential adjunct to the treatment of mental conditions, that are either marked by impairments in memory performance or where treatments are hinged upon successful learning of new information. In the following sections we will highlight such clinical areas, where taVNS may therefore be utilized to aid treatment strategies, including neurocognitive, anxiety and trauma-related disorders (see Figure 2).

### Neurocognitive disorders

It is well established, that episodic and associative memory performance declines with increasing age (Shing et al., 2010; Greene and Naveh-Benjamin, 2020) and such memory decline has found to be partly grounded in LC integrity (Dahl et al., 2019; Dahl et al., 2023) and overall neurodegenerative actions (Maass et al., 2018). Since VNS specifically targets the LC, but also increases overall cortical and hippocampal neuroplasticity (Biggio et al., 2009; Morrison et al., 2019; Keute and Gharabaghi, 2021), and invokes memory-enhancing effects (Ventura-Bort et al., 2021), it may be presumed that VNS might counter such deterioration in ageing individuals. In fact, taVNS has found to improve both episodic and associative memory in elderly people (Jacobs et al., 2015, see Table 1).

However, in some cases age-related impairments in memory performance are particularly pronounced: Mild cognitive impairment (MCI) describes such a condition of cognitive decline, which is has been conceptualized as an intermediate point between normal ageing and dementia (Simon et al., 2012). At this, MCI either includes memory impairments (amnestic subtypes) or not (non-amnestic subtypes) (Bradfield, 2023). Importantly, amnestic MCI is highly present among elderly people, with studies reporting prevalence rates ranging from 0.5% up to 31.9% (median 4.9%) (Ward et al., 2012). To combat memory impairments in amnestic MCI, current treatments often build upon drugs like cholinesterase inhibitors (e.g., galantamine; Loy and Schneider, 2006) or cognitive interventions, such as visual imagery, chunking or cueing (Simon et al., 2012). However, given that MCI has found to be linked to reduced locus coeruleus integrity (Jacobs et al., 2021), such treatments could be complemented by LC-targeting taVNS, effectively utilizing its memory-enhancing effects: Indeed, preliminary data suggests that VNS triggered alterations in functional connectivity between memory-relevant brain regions (Murphy et al., 2023) and demonstrated to improve both the establishment of associative memory as well as immediate and delayed episodic memory recall in MCI patients (Wang et al., 2022; Dolphin et al., 2023).

Longitudinal studies indicated, that amnestic MCI is a critical risk factor for the development of Alzheimer’s disease (AD), as it progresses to AD at an average rate of 10%–17% per year (Ferman et al., 2013). This is not surprising, given that amnestic MCI and AD share common features of neurodegeneration and memory decline (Weller and Budson, 2018). According to the World Alzheimer Report 2018, it was estimated that about 50 million people worldwide suffer from AD, and it was projected that this prevalence will triple by the year of 2050 (Patterson, 2018). To combat the progredient memory loss in AD, effective treatments currently tap into similar mechanisms as therapeutic strategies for amnestic MCI: Drugs, such as cholinesterase inhibitor galantamine and NMDA-antagonist memantine, complemented by cognitive interventions (Loy and Schneider, 2006; Weller and Budson, 2018). Given the striking similarities between amnestic MCI and AD, it might be presumed that VNS could provide a valuable addition to this list. Indeed, first pilot studies suggest that taVNS might alleviate AD symptomatology (Sjogren et al., 2002; Merrill et al., 2006) and these effects are further tested in currently ongoing clinical trials (Vargas-Caballero et al., 2022).

### Anxiety and trauma-related disorders

Besides neurocognitive disorders, memory processes also play a pivotal role in the etiology and treatment anxiety and trauma-related disorders–conditions, that share features of excessive fear-based symptoms elicited by distinct (trauma-related) fear cues (American Psychiatric Association, 2013). Pavlovian fear conditioning is widely regarded as a model for the establishment of such excessive fear (Craske et al., 2006). In contrast, fear extinction is considered to be an associative learning mechanism driving the success of exposure-based cognitive behavioral therapy–the current first-line treatment for anxiety and trauma-related disorders (Craske et al., 2014; Carpenter et al., 2018). Such treatment involves the repeated exposure towards the patient’s individual fear cues, invoking extinction learning which ultimately results in a gradual inhibition of fear-based symptoms (Craske et al., 2014). However, as indicated by basic research, extinction memory is rather fragile compared to the original fear memory trace (Dunsmoor et al., 2015b), and thus the organism remains prone to fear memory reactivation. Such proneness might be even more pronounced upon deficient extinction, which thus constitutes a risk factor for non-responding to treatment or relapses (Vervliet et al., 2013; Milad et al., 2014). Importantly, extinction deficits are prevalent in anxiety and trauma-related disorders, possibly contributing to high rates of non-responders (∼50%) and relapses (∼14%) in exposure-based treatments (Loerinc et al., 2015; Levy et al., 2021). Given the positive effects of both invasive and non-invasive VNS on the formation and recall of extinction memory in animals and humans (see above), VNS might constitute a valuable adjunct to overcome these deficits and boost the success of exposure-based therapeutic strategies.

In fact, first evidence on invasive VNS supports this view: In a recent study of Powers and colleagues (Powers et al., 2025), iVNS was combined with prolonged exposure therapy in treatment-resistant PTSD. By applying the invasive VNS during twelve treatment sessions, PTSD symptoms were indeed substantially alleviated–an effect, which persisted even 6 months after the cessation of therapy and ultimately resulted in a loss of PTSD diagnosis in all participants.

Non-invasive taVNS, which may be viewed as a more applicable alternative to iVNS due to not requiring surgery, results in similar effects: In a study of Szeska et al. (2025), spider phobic individuals underwent a standardized exposure *in vivo* towards a living tarantula, which was followed by a complementary taVNS-paired laboratory exposure *in vitro*, during which participants were presented with pictures of various spiders including the exposed specimen. Participants, that received active VNS during this complementary exposure indeed showed a stimulus-specific inhibition of fear responses, as indexed by attenuated fear tachycardia and corrugator activity towards pictures of the exposed tarantula–autonomic and behavioral components of fear, that serve as indirect read-outs of amygdala activity (Heller et al., 2014; Roelofs and Dayan, 2022). This effect became stronger with increasing stimulation duration, indicating a dose-dependency of stimulation effects. Importantly, fear attenuation even maintained after stimulation had ceased and participants were subjected to a second *in vivo* exposure: After receiving taVNS, participants were more likely to make physical contact with the phobic stimulus and touch the tarantula with bare hands, as opposed to participants that received a sham stimulation of the earlobe. Together, these results indicate that vagus nerve stimulation boosts a stimulus-specific reduction of fear responses in a dose-dependent manner, which culminates in promoted responding to exposure treatment.

In sum, current evidence therefore suggests that VNS might be a powerful adjunct for therapeutic regimen that build upon exposure effects (Craske et al., 2014), including first-line treatments of anxiety, stressor-and trauma-related disorders, as well as obsessive-compulsive disorder.

## Discussion

A prioritized storage of emotionally salient stimuli into long-term memory warrants that significant cues, people, places and events can be remembered in the future, thus acting as a prerequisite for behavioral adaptivity (Dunsmoor and Kroes, 2019). Animal research has long suggested the vagus nerve to be a central relay driving this effect, since adrenergic actions on this nerve in emotionally arousing situations elicit central noradrenergic release, which promotes plasticity in memory-relevant brain areas (McIntyre et al., 2012). The availability of electrical invasive and non-invasive vagus nerve stimulation (VNS) has recently opened up the possibility to test a comparable vagal route to memory in humans. In this review, we synthesized animal and human studies utilizing VNS, which altogether suggested that (1) the vagus nerve constitutes an evolutionarily preserved brain-body axis driving emotional memory formation and (2) VNS may consequently be used to promote emotional episodic and associative memory consolidation. Following up on the latter notion, we further discussed VNS as a tool to combat memory decline in neurocognitive disorders and as an adjuvant to facilitate learning processes underlying exposure-based treatment of anxiety and trauma-related disorders. In fact, preliminary evidence suggests that electrical VNS improves mnemonic performance in mild cognitive impairment and Alzheimer’s disease (Wang et al., 2022), just as it promotes responding to exposure-based treatment (Powers et al., 2025; Szeska et al., 2025), rendering electrical VNS as a promising adjunct to a variety of therapeutic strategies. Interestingly, this might also apply to non-electrical VNS: The afferent vagus nerve is a critical component of the microbiota-gut-brain axis and thus it may be possible to invoke VNS by manipulations of the microbiome to achieve similar beneficial effects (Kuijer and Steenbergen, 2023; Faraji et al., 2025).

Nevertheless, more research into the mechanisms of electrical VNS is necessary to fully exhaust its memory-enhancing potential and utilize this stimulation technique in clinical areas: Although there is abundant evidence that VNS increases noradrenergic transmission by projections to the locus coeruleus (Frangos et al., 2015; Yakunina et al., 2017; Ventura-Bort et al., 2018; Sclocco et al., 2019; Borgmann et al., 2021; Teckentrup et al., 2021; Giraudier et al., 2022), VNS also targets centers of other transmitter systems (Frangos et al., 2015) and thus modulates cholinergic (Bowles et al., 2022), dopaminergic (Manta et al., 2009; Brougher et al., 2021), serotonergic (Manta et al., 2009, 2013) as well as glutamatergic and GABA-ergic neural transmission (Ben-Menachem et al., 1995). As each of these systems is involved in memory formation (Myhrer, 2003), the exact mechanism that underlies VNS-driven memory enhancement is yet to disentangle. Likewise, it needs to be determined under which conditions VNS yields the strongest memory-enhancing effects: Of the 40 articles included in our mini-review (see Table 1), only four have reported null-effects by VNS, with all using taVNS. Thus, we may preliminarily conclude that iVNS produces more consistent effects, possibly as it exhibits more direct action on the vagus. However, future studies need to additionally test the role of different stimulation parameters (frequency, intensity, duration; see D’Agostini et al., 2023) in VNS effects: Indeed, it has been shown that adrenergic agents affect memory retention in an inverted-U-shaped function, that depends upon the arousal level of the organism, where exceeding levels of systemic adrenaline may even impair memory performance (Gold and Korol, 2012). As VNS is suggested to tap into similar mechanisms, it is tempting to speculate that its memory enhancing effects are similarly shaped, i.e., being strongest at intensities that – when acting upon the current arousal state – invoke moderate levels of adrenergic activation (for preliminary evidence, see Souza et al., 2021). However, besides the current arousal state, further individual characteristics may also shape VNS effects and need to be systematically investigated (e.g., age, genetic factors, baseline cognitive functions, but also baseline vagal tone). Furthermore, it needs to be tested how (1) acute vs. multiple stimulation, (2) online (during task) vs. offline stimulation (before and/or after task), (3) type of task (e.g., relying on HC function), and (4) immediate vs. delayed testing impact on the effects of VNS on learning and memory. This also applies to the affective valence of the encoded material: While our review showed that VNS enhances memory for unpleasant material, to best of our knowledge there is no study that investigated whether the same holds true for pleasant stimuli, despite (mnemonic) processing similarly taps into (nor-) adrenergic mechanisms (Sternberg et al., 1985; Codispoti et al., 2003). Hence, we hope that our mini-review strongly encourages specific experimental designs or meta-and mega-analytic approaches (see Giraudier et al., 2022) to answer these open research questions. While VNS is already an FDA-approved clinical treatment of epilepsy and drug-resistant major depressive disorder (and applied in further clinical trials focusing e.g., on Alzheimer’s disease, mild cognitive impairment, PTSD, alcohol use disorder or stroke rehabilitation; see (Herr et al., 2024)), addressing these gaps will help to integrate it even more effectively into therapeutic strategies and tailor this stimulation technique for specific patient populations.

Altogether, this mini-review revealed that the vagus nerve constitutes a major communication route between the body’s periphery and the brain, which is critically involved in the formation of emotional memories. Vagus nerve stimulation can therefore be considered as one of the most promising neuromodulation techniques to combat mental disorders, and its full potential at this is yet to unfold.
